# BKV Related Hemorrhagic Cystitis—An Insight into Risk Factors and Later Complications—An Analysis on Behalf of Polish Adult Leukemia Group

**DOI:** 10.3390/cancers14030764

**Published:** 2022-02-01

**Authors:** Jarosław Dybko, Agnieszka Piekarska, Siddarth Agrawal, Sebastian Makuch, Donata Urbaniak-Kujda, Monika Biernat, Blanka Rybka, Magdalena Dutka, Alicja Sadowska-Klasa, Sebastian Giebel, Lidia Gil

**Affiliations:** 1 Department of Hematology and Cellular Transplantation, Lower Silesian Oncology Center, 53-413 Wroclaw, Poland; 2 Department of Hematology and Transplantology, Medical University of Gdansk, 80-214 Gdansk, Poland; agnieszka.piekarska@gumed.edu.pl (A.P.); magdalena.dutka@gumed.edu.pl (M.D.); alicja.sadowska-klasa@gumed.edu.pl (A.S.-K.); 3 Department and Clinic of Internal Medicine, Occupational Diseases, Hypertension and Clinical Oncology, Wroclaw Medical University, 50-556 Wroclaw, Poland; siddarth@agrawal.pl; 4 Department of Clinical and Experimental Pathology, Wroclaw Medical University, 50-368 Wroclaw, Poland; sebastian.makuch@student.umw.edu.pl; 5 Department of Hematology and Bone Marrow Transplantation, Wroclaw Medical University, 50-367 Wroclaw, Poland; donata.urbaniak-kujda@umw.edu.pl (D.U.-K.); monika.biernat@umw.edu.pl (M.B.); 6 Department of Pediatric Bone Marrow Transplantation, Oncology, and Hematology, Wroclaw Medical University, 50-556 Wroclaw, Poland; blanka.rybka@umw.edu.pl; 7 Department of Bone Marrow Transplantation and Hematology-Oncology, National Research Institute of Oncology, 44-102 Gliwice, Poland; sebastian.giebel@io.gliwice.pl; 8 Department of Hematology and Bone Marrow Transplantation, Poznań University of Medical Sciences, 60-569 Poznan, Poland; lidia.gil@skpp.edu.pl

**Keywords:** BK virus, allogeneic hematopoietic stem cell transplantation, risk factors, viremia

## Abstract

**Simple Summary:**

Despite noticeable progress in allogeneic hematopoietic stem cell transplantation, potential viral reactivations are still one of the most challenging complications. The aim of our study was to identify predictive and risk factors associated with the occurrence of the BK virus related hemorrhagic cystitis following hematopoietic stem cell transplantation. Furthermore, we investigated the impact of various factors on the clinical course of patients after hematopoietic stem cell transplantation. We confirmed that >0.75 × 10^3^ BK virus copies/mL in serum at day +21 after allogeneic hematopoietic stem cell transplantation has a strong predictive ability for hemorrhagic cystitis. Thus, we believe that our findings could be helpful in establishing the predictive validity of the BK viral load measurement in polyomavirus BK-associated hemorrhagic cystitis and survival.

**Abstract:**

BK virus reactivation increases the likelihood of hemorrhagic cystitis (HC) after allogeneic hematopoietic stem cell transplant (HCT). In this study, we aimed to identify predictive and risk factors associated with the increased occurrence of this condition following HCT. On a group of 124 patients aged ≤71 years old (median 40 years) who underwent HCT, we analyzed sex, age, time from diagnosis to transplantation, type of conditioning, donor’s relationship, age, and sex, the impact of immunosuppression with different drugs, and acute and chronic GVHD, BK viremia and viruria as potential factors increasing the risk of BK-related HC after HCT. HC occurred among 24 patients (24/124; 29.2%). A significant correlation was observed between HC incidences after HCT, BK viremia and viruria, and acute GVHD occurrence. Furthermore, the level of BKV DNA in serum at day +21 (>0.75 × 10^3^) significantly impacted the patients’ survival time. According to our results, the likelihood ratio of BKV-DNA on day +21 in serum is 6.25, indicating that this diagnostic test has the potential to be utilized in a clinical setting. These findings may be used as a voice in the discussion on implementing an optimal preemptive treatment in BKV reactivation after allogeneic HCT.

## 1. Introduction

The BK virus (BKV)-associated hemorrhagic cystitis (BK-PyVHC) is a well-recognized complication and a common cause of morbidity in patients undergoing hematopoietic cell transplantation (HCT) [1]. Besides increasing the morbidity of HCT recipients, BKV prolongs hospital stay, increases the risk of death, and escalates healthcare costs [2]. BKV reactivation following allogeneic HCT can lead to manifestations ranging from asymptomatic viruria to severe hemorrhagic cystitis (HC) [3]. When the BK virus reactivates in immunocompromised hosts (such as HCT or solid organ transplant recipients) or immunocompromised (such as AIDS patients), it can lead to severe clinical diseases. For example, BKV reactivation can lead to polyomavirus-associated nephropathy in kidney transplant patients [4,5]. Additionally, BKV reactivation may result in renal failure and allograft failure in kidney transplant patients. Several studies have attempted to recognize risk factors associated with BKV-HC [6,7,8,9]. High-level viruria and/or the presence of viremia have been associated with the diagnosis of BKV-HC [3,10]. Other risk factors identified and published by the European Conference on Infectious Disease in Leukemia (ECIL) for BK-PyVHC include acute graft-versus-host disease (GVHD), type of conditioning, stem cell source, type of donor, and age >7 years old [10]. In our study, we attempted to identify predictive and risk factors associated with the occurrence of BKV related HC following HCT. Furthermore, we investigated the impact of various factors on the clinical course of patients after HCT.

## 2. Materials and Methods

The study involved 124 patients transplanted between 2015 and 2017 in 4 Polish centers. The whole cohort consisted of 52 women (41.9%) and 72 men (58.1%) aged ≤71 years old (median *Me* = 40 years), who underwent allogeneic stem cell transplantation from matched unrelated or sibling donor. Twenty-four of them developed HC (HC group), and 100 were free of this complication (non-HC group). All HC patients were treated with forced hydration and spasmolytics; in 15 cases continuous bladder irrigation was introduced (8 just with saline solution, 7 additionally with sodium hyaluronate). Hyperbaric oxygen therapy was used in 12 cases. Seventeen patients required systemic therapy with a conventional once a week Cidofovir dose (5 mg/kg b.w from 2 up to 4 doses). Eighty-four patients of the whole group were diagnosed with acute leukemia (AML-57, ALL-27), 20—myelodysplastic syndromes, 10—myelofibrosis, and 10 with different types of lymphoma (including 3 myeloma cases). Thirty-four patients died in the whole group: 16 due to original disease relapse, 4—direct HC complications, 6—infectious complications other than BKV, 8—acute GvHD. The study was designed as retrospective with serum and urine BKV-DNA monitoring on +14, +21, and +28 as a part of the protocol. We analyzed: (a) diagnosis; (b) sex age at the time of transplantation; (c) time from diagnosis to transplantation; (d) conditioning (MAC vs. RIC); (e) total body irradiation (TBI) as a part of conditioning; (f) the number of CD34 cells and the source of cells (bone marrow (BM) vs. peripheral blood stem cells (PBSC); (g) donors’ demographics; (h) donors’ origin (matched unrelated donor (MUD) vs. non-MUD); (i) the impact of immunosuppression with cyclosporine (CsA), methotrexate (MTX) and mycophenolate mofetil (MMF) as GvHD prophylaxis; (j) serum and urine BKV-DNA level; (k) acute GVHD; (l) chronic GVHD;—as potential risk factors associated with complications caused by BKV following HCT.

### Statistical Analysis

The distribution of the variables was assessed using descriptive statistics. The quantitative empirical distributions of variables were verified in terms of their normality using the Shapiro–Wilk test. Due to the fact that they differed significantly from the normal distribution, non-parametric tests (Mann–Whitney) were used in further analysis. Independent *t*-tests evaluated the mean differences by participants’ characteristics. The Kaplan–Meier method was used to estimate patients’ probability of survival. ROC analysis was used for evaluating the discriminatory performance of a continuous variable. Likelihood ratios have been used as a tool to evaluate the potential utility of BKV-DNA Day +21 serum as a diagnostic test. All analyses were performed using the statistical software package Statistica. A *p*-value of < 0.05 was considered to be statistically significant.

## 3. Results

### 3.1. BKV-DNA PCR Analysis

**Figure 1:**
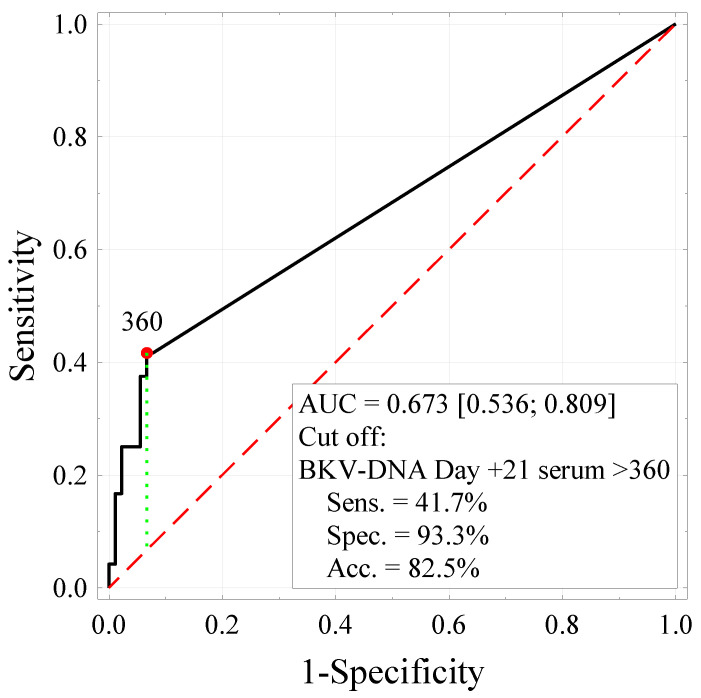
ROC curve analyzing the time from diagnosis and the level of BKV-DNA in serum on day +21 (×10^3^), showing the diagnostic test’s sensitivity, specificity, and accuracy.

**Figure 2:**
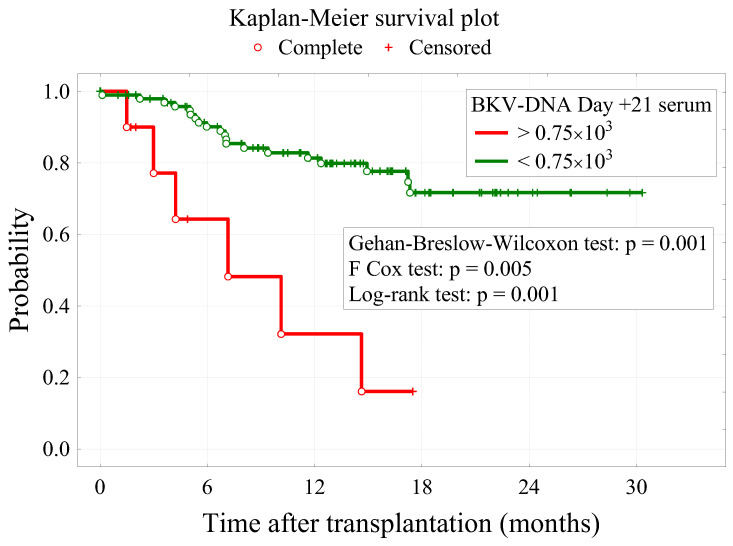
Kaplan–Meier survival plots of patients differing in the level of BKV-DNA in serum on day +21.

BKV-DNA levels were significantly higher on days +14 and +21 in serum (*p* = 0.027 and *p* < 0.001 respectively) and on days +14, +21, and +28 in urine (*p* < 0.001 in all three cases) in the HC group. The difference in BKV-DNA on day 28 in serum (×10^3^) was not statistically significant (*p* = 0.153). We found that the level of BKV DNA in serum on day +21 (<0.75 × 10^3^) (*p* = 0.049) had a significant impact on the patients’ survival time. The cut-off values for the BKV DNA on day +21 in serum factor were determined based on ROC curves analysis (Figure 1). Patients with BKV-DNA levels higher than 0.75 × 10^3^ were at a significantly higher risk of death (Figure 2). To verify the potential utility of diagnostic testing of the serum BKV-DNA on day +21 in early detection of complications, such as BKV-related HC following HCT, we used the likelihood ratios as a reliable tool to examine the sensitivity and the specificity of the BKV DNA test. Considering that the likelihood ratio of 5–10 are highly reliable and the fact that, according to our results, the likelihood ratio of BKV-DNA on day +21 in serum is 6.25 (Table 1), we can clearly state that this diagnostic test is reliable and may be utilized in a clinical setting.

### 3.2. GvHD and Other HC Risk Factors

We compared the analyzed risk factors potentially associated with HC. There was no statistically significant difference between the HC and non-HC group for the following variables: sex (*p* = 0.237); age (*p* = 0.304); MAC conditioning (*p* = 0.932); TBI in the conditioning (*p* = 0.906); number of CD34 cells (*p* = 0.227); MUD donor (*p* = 0.874); donor’s age (*p* = 0.273); donor’s sex (*p* = 0.609); chronic GVHD (*p* = 0.139). However, the incidence of acute GvHD was significantly higher in the HC group (*p* = 0.021) and, as expected, the results showed that acute GVHD was significantly more frequent in the HC group (66.7% vs 38.0% in non-HC group, *p* = 0.021).). The analyzed risk and prognostic factors associated with HC and the clinical characteristics of patients are shown in Table 2. A multivariate logistic regression analysis showed that BKV-DNA at day +21 in serum and acute GvHD are statistically significant risk factors for HC (Table 3).

### 3.3. Survival Analysis

**Figure 3:**
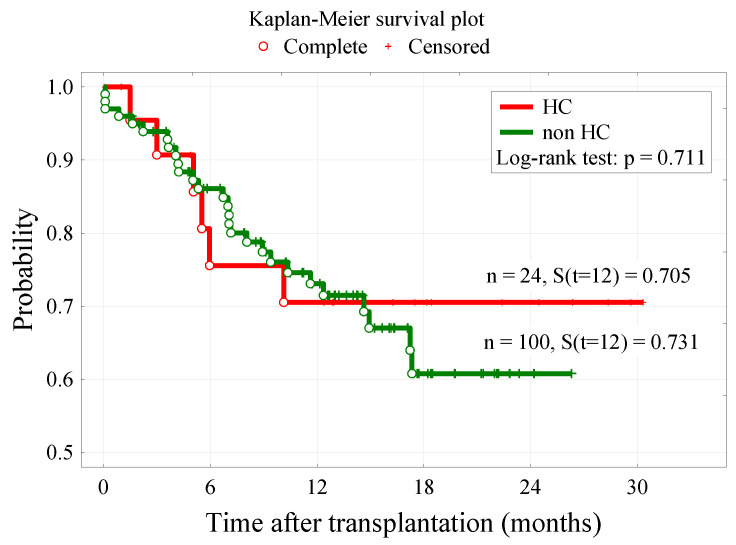
Kaplan–Meier survival plots of patients in HC and non-HC groups.

There was no difference between groups in overall survival (Figure 3), but as we mentioned before, the level of BKV DNA in serum on day +21 (<0.75 × 10^3^) (*p* = 0.049) had a significant impact on the patients’ survival time. Moreover, a longer time from diagnosis to transplantation (*p* = 0.017), and conditioning other than MAC (*p* = 0.028), were associated with higher mortality. Furthermore, other factors that were found to have a significant impact on patients’ survival were MAC conditioning (*p* = 0.028) and time from diagnosis to transplantation (*p* = 0.017). Surprisingly, better survival was associated with MAC conditioning than RIC (reduced-intensity conditioning). Patients who underwent HCT within 12 months after the diagnosis had a significantly better prognosis. A detailed characteristic of patients based on survival is presented in Table 4. A multivariate logistic regression analysis showed that MAC conditioning is the only statistically significant risk factor for survival (Table 5).

## 4. Discussion

Despite noticeable progress in allogeneic HCT, potential viral reactivations are still one of the most challenging complications. Among the polyomaviruses, BK virus reactivation is the most common one [6] and increases the risk of late-onset HC incidences (occurring mainly between 2 and 8 weeks (range one week–6 months) after HCT) [10]. According to our study on a total of 124 patients, HC complications caused by BK infection were reported in 24 patients (24/124; 29.9%). This value is within the average rates of BKPyV-HC incidences, as reported in ECIL guidelines [10] (8–25% and 7–54% in pediatric and adult patients, respectively). The current literature highlights a variety of risk factors for HC following allo-HCT, including myeloablative (MAC) conditioning, unrelated donors, other than BK-HC associated viremias such as cytomegalovirus (CMV) or human herpesvirus (HHV)-6, and graft-versus-host disease (GVHD) [11,12,13]. However, all these factors were not observed consistently in independent studies. For instance, Giraud et al. determined that unrelated donors are associated with higher cumulative HC incidences compared to related donors [8]. These results are contrary to our finding; we did not find significant correlations between the donor origin. The number of HC cases in the cohort is similar with Giraud’s and our study but our group consists of approximately 25% of sibling donor HCT, while in the Giraud/s study it is almost 40%. Similarly, Giraud et al. found MAC conditioning as a risk factor of BKV-associated HC after allo-HCT and we did not find any significant difference between conditioning regimens. While analyzing the data we report only 35% of non-MAC cases while in Giraud’s study it is more than 60%. In both studies TBI was used in almost 50% of MAC cases. In multiple studies, a strong association between BK-PyVHC and acute GVHD was observed [12,14] and our findings, confirmed in a multivariate analysis, are compatible. Although several studies failed to show any correlation between acute GVHD and HC [15], most of the studies indicated a significant association between these two HCT-related complications [16,17,18], which is confirmed in our study. Additionally, high dose corticosteroids may attenuate lymphocyte recovery, which results in inaccurate immunological surveillance, and finally higher viremia and increased risk of HC [19]. In our study, in the HC group, corticosteroids were used significantly more frequently (*p* = 0.027). Moreover, we did not find any significant correlation between age and HC in transplant patients (*p* = 0.304), which is contrary to other studies [18,20] but corresponding with a large study by Uhm et al. [16].

Furthermore, we determined that the level of BKV-DNA on days 14, 21, and 28 in urine (×10^9^ copies/mL) (*p* < 0.001 in all three cases) is associated with an increased risk of HC in transplant patients. This finding is in line with Hayden et al., who noticed that recipients with a peak urinary BK virus load ≥1 × 10^9^ copies/mL developed HC approximately seven times more often than those with a peak viral load <1 × 10^9^ copies/mL; a peak BK viruria was seen in a median of 13 days prior to the occurrence of HC [18]. Studies of Bogdanovic et al. and Cesaro et al., on the other hand, suggested a slightly lowered cut-off viral load—10^6^ BKV copies/mL in urine—as a predictive factor for HC in patients after HCT. BK viruria occurred, on average, 13 days and 18 days before HC, respectively [17]. It is worth keeping in mind that the BK virus may be isolated from serum or/and urine of asymptomatic patients before HCT and even in healthy individuals. It was determined that approximately more than 80% of all HCT patients develop high levels of BK viruria, while only 5–20% progress to BKV-associated HC [21,22], suggesting that late-onset HC requires cofactors other than BK viruria for its development. For instance, Cesaro et al. found that among 15 HCT patients who were monitored for BK virus, five (33%) of them developed HC. Including non-HC, healthy patients, three were positive for BKV in the urine [23]. Gaziew et al. also found a group of 10 patients with peak BK viruria of more than 55 × 10^6^ copies/mL who did not develop HC [16]. Furthermore, Leung et al. measured BKV viruria in 50 patients after bone marrow transplantation; only 40% of them developed HC [24]. As suggested by Leung et al., intensive immunosuppression after transplantation may increase viral replication and, in consequence, its reactivation. When the viral reactivation occurs at a low level, patients are asymptomatic. However, when viral replication exceeds a certain level (as 10^9^ copies/mL according to our study), the BK virus causes uroepithelial cell lysis, resulting in HC [24].

Our study also indicated that the level of BKV-DNA on days 14 and 21 in serum (×10^3^ copies/mL) (*p* = 0.027 and *p* < 0.001, respectively) was associated with increased risks of HC in HCT patients. Furthermore, our study revealed that the level of BKV-DNA on days +21 in serum after allo-HCT is an independent, predictive survival factor. If the number of viral copies/mL in serum 21 days after allo-HCT exceeds ≥0.75 × 10^3^, the risk of death increases significantly. Cesaro et al. assessed the value of plasma BKV load in 15 pediatric patients to predict HC. They determined that the BKV load of 10^3^ copies/mL in plasma was significantly associated with HC (*p* = 0.0006), which is in concordance with our finding (≥0.75 × 10^3^ copies/mL). More importantly, they also determined that the increase of BKV load of 10^3^ copies/mL in plasma is usually observed on day 17 before HC becomes clinically manifest [23]. This finding was also confirmed in a study aiming to predict HC after HCT by determining BKV viremia load in plasma. It has been shown that a plasma BKV load of 1 × 10^3^ copies/mL had the best sensitivity and specificity in predicting HC within 2 weeks before its clinical appearance [25]. In accordance with this result, Ghosh A et al. determined that out of 224 consecutive HCT patients, BKV-related HC occurred in 25 of them (11.2%) at a median of 32 days after HCT [26].

Including the fact that BK viruria and viremia may be observed in asymptomatic HCT patients, their screening is currently not recommended by ECIL guidelines [10], as preemptive therapy is not established. Nevertheless, our findings show that monitoring BK viremia loads at day +21 of transplantation is possibly important as a predictive factor in patients treated with allogeneic HCT. Recently, Laskin et al. tested the associations of peak BKV viremia ≥10,000 with overall survival at 2 years after HCT in children and young adults. Again, viremia ≥10,000 copies/mL was associated with a higher risk of death, regardless of whether the patient was symptomatic or not [12]. In addition, Imlay et al. indicated that high plasma BKPyV load at the beginning of BKPyV-HC (≥10,000 copies/mL) was significantly associated with longer durations of hematuria and other HC-related symptoms [3]. Thus, we believe that our findings could be helpful in establishing the predictive validity of BKV viral load measurement in BKPyV-HC and survival. Further trials identifying risk factors associated with increased mortality in HC patients undergoing allogeneic HCT are required to establish a possible prophylactic approach in this complication.

## 5. Conclusions

BKV infection is a common complication of HCT, associated with significant and prolonged morbidity, especially in the setting of acute GVHD, MAC conditioning, long timing from diagnosis to transplantation, and high-grade BK viremia and viruria loads. This study indicates that >0.75 × 10^3^ BKV copies/mL in serum at day +21 after allo-HCT has a strong predictive ability for HC. Prospective studies are needed to further define HC morbidity, identify risk factors and inform the design of prophylaxis and treatment trials for HC conditions after allogeneic HCT.

## Figures and Tables

**Table 1:** The likelihood ratios of BKV-DNA viremia and viruria.

Factor	Cut-Off	Sens.	Spec.	Acc.	LR(+)	AUC
BKV-DNA Day +14 serum	≥0.098 × 10^3^	22.7%	93.8%	80.5%	3.64	0.580 (0.437–0.722)
BKV-DNA Day +14 urine	≥0.036 × 10^9^	65.0%	85.9%	82.1%	4.60	0.752 (0.623–0.881)
BKV-DNA Day +21 serum	≥0.36 × 10^3^	41.7%	93.3%	82.5%	6.25	0.673 (0.536–0.809)
BKV-DNA Day +21 urine	≥0.001 × 10^9^	70.8%	73.3%	72.8%	2.65	0.728 (0.609–0.848)
BKV-DNA Day +28 serum	≥0.098 × 10^3^	28.6%	87.5%	75.2%	2.29	0.568 (0.426–0.709)
BKV-DNA Day +28 urine	≥0.03 × 10^9^	57.9%	86.5%	80.6%	4.28	0.707 (0.566–0.848)

**Table 2:** Clinical characteristics of patients that underwent allogeneic stem cell transplantation—factors associated with the occurrence of hemorrhagic cystitis (HC).

Variable	HC		*p*-Value
	Yes *n* = 24	No *n* = 100	
Female, *n* (%)	7 (29.2%)	45 (45.9%)	0.237
Median age at transplant (years), Me (Q1; Q3)	39 (31; 52)	46 (30; 58)	0.304
Time from diagnosis to transplantation (months) [Q1:Q3]	7 (6; 20)	11 (6; 21)	0.473
Time from HC manifestation following HSCT (days)	22.5 (14; 30.25)	-	-
Conditioning MAC	16 (66.7%)	65 (65.0%)	0.932
TBI	8 (33.3%)	32 (32.0%)	0.906
CD34 [Q1:Q3]	5.8 (4.7; 6.7)	6.0 (5.1; 7.5)	0.227
PBSC	20 (83,3%)	92 (92%)	0.591
Donor MUD	18 (75.0%)	74 (74.0%)	0.874
Age, donor (Q1:Q3)	25 (6; 35)	9 (4; 35)	0.273
Corticosteroids	16 (67%)	36 (36%)	**0.027**
BKV-DNA Day +14 serum (×10^3^) (Q1:Q3)	0.00 (0.00; 0.00)	0.00 (0.00; 0.00)	**0.027**
BKV-DNA Day +14 urine (×10^9^) (Q1:Q3)	0,14 (0.00; 4,48)	0.00 (0.00; 0.00)	**<0.001**
BKV-DNA Day +21 serum (×10^3^) (Q1:Q3)	0.00 (0.00; 1,07)	0.00 (0.00; 0.00)	**<0.001**
BKV-DNA Day +21 urine (×10^9^) (Q1:Q3)	0.42 (0.00; 1,68)	0.00 (0.00; 0.00)	**<0.001**
BKV-DNA Day +28 serum (×10^3^) (Q1:Q3)	0.00 (0.00; 0,10)	0.00 (0.00; 0.00)	0.153
BKV-DNA Day +28 urine (×10^9^) (Q1:Q3)	0.07 (0.00; 5.40)	0.00 (0.00; 0.00)	**<0.001**
Acute GvHD	16 (66.7%)	38 (38.0%)	**0.021**
Chronic GvHD	11 (45.8%)	30 (30.0%)	0.139
CMV	17 (71%)	42 (46.7%)	0.126
CMV, donor	9 (38%)	48 (48%)	0.322
Dead	6 (25.0%)	28 (28.0%)	0.967
Survival (months) (Q1:Q3)	12.9 (5.0; 20.4)	11.2 (5.5; 16.3)	0.374

Quantitative variables were presented as medians and quartile ranges (Q1; Q3). Values are number of cases, with percent in parentheses, unless otherwise specified. MUD—matched unrelated donor; TBI—total body irradiation; MAC—myeloablative conditioning. Bold values denote statistical significance at *p* < 0.05 level.

**Table 3:** Results of multivariate logistic regression analysis of HC risk.

HC Risk Factor	*b*	SE_b_	*p*	OR (95% CI)
BKV-DNA Day +14 serum	3.2 × 10^−4^	2.4 × 10^−4^	0.181	-
BKV-DNA Day +14 urine	−1.6 × 10^−12^	5.8 × 10^−12^	0.780	-
BKV-DNA Day +21 serum	4.0 × 10^−4^	1.5 × 10^−4^	**0.007**	1.0004 (1.0001–1.0007)
BKV-DNA Day +21 urine	7.5 × 10^−11^	6.4 × 10^−11^	0.237	-
BKV-DNA Day +28 urine	−8.0 × 10^−12^	1.5 × 10^−12^	0.584	-
Acute GvHD	1.332	0.533	**0.012**	3.787 (1.332–10.765)

**Table 4:** Clinical characteristics of patients that underwent allogeneic stem cell transplantation—factors associated with survival.

Variable	Status		*p*-Value
	Dead *n* = 34	Alive *n* = 90	
Female, *n* (%)	11 (32.4%)	41 (45.6%)	0.184
Age (years), Me [Q1; Q3]	48 [34; 58]	43 [30; 55]	0.190
Time from diagnosis to transplantation (months) [Q1; Q3]	18 [8; 44]	9 [6; 18]	**0.017**
Conditioning MAC	17 (50.0%)	64 (71.1%)	**0.028**
TBI	11 (32.4%)	29 (32.2%)	0.989
CD34 [Q1; Q3]	6.2 [4.9; 7.3]	6.0 [5.2; 7.3]	0.606
PBSC	30 (88.2%)	82 (91.1%)	0.523
Donor MUD	27 (79.4%)	65 (72.2%)	0.558
Age, donor [Q1; Q3]	14 [4; 36]	9 [4; 34]	0.573
Female, donor	12 (35.3%)	24 (26.7%)	0.345
HLA	29 (85.3%)	83 (92.2%)	0.410
BKV-DNA Day +14 serum (×10^3^) [Q1; Q3]	0.00 [0.00; 0.00]	0.00 [0.00; 0.00]	0.525
BKV-DNA Day +14 urine (×10^9^) [Q1; Q3]	0.00 [0.00; 0,11]	0.00 [0.00; 0,02]	0.547
BKV-DNA Day +21 serum (×10^3^) [Q1; Q3]	0.00 [0.00; 0.36]	0.00 [0.00; 0.00]	**0.049**
BKV-DNA Day +21 urine (×10^9^) [Q1; Q3]	0.00 [0.00; 0,11]	0.00 [0.00; 0.50]	0.991
BKV-DNA Day +28 serum (×10^3^)	0.00 [0.00; 0.00]	0.00 [0.00; 0.00]	0.848
BKV-DNA Day +28 urine (×10^9^) [Q1; Q3]	0.00 [0.00; 0.16]	0.00 [0.00; 0.00]	0.556
Acute GvHD	14 (41.2%)	40 (44.4%)	0.743
Chronic GvHD	8 (23.5%)	33 (36.7%)	0.241
CMV	17 (50%)	42 (46.7%)	0.319
CMV, donor	16 (47%)	41 (45.6%)	0.422
HC	6 (17.6%)	18 (20.0%)	0.967

Quantitative variables were presented as medians and quartile ranges [Q1;Q3]. Values are number of cases, with percent in parentheses, unless otherwise specified. Bold values denote statistical significance at *p* < 0.05 level.

**Table 5:** Results of multivariate logistic regression analysis of death risk.

Death Risk Factors	*b*	SE_b_	*p*	OR (95% CI)
Time from diagnosis to transplantation (months)	0.007	0.005	0.198	1.00 (0.996–1.018)
Conditioning MAC	−0.885	0.415	0.033	0.415 (0.183–0.930)
BKV-DNA Day +21 serum	1.8 × 10^−4^	1.3 × 10^−4^	0.167	1.00 (0.9999–1.0004)

## Data Availability

The data presented in this study are available on request from the corresponding author. The data are not publicly available due to ethical considerations.
